# The Enlargement of Small Hospitals

**Published:** 1920-01-10

**Authors:** 


### HOSPITAL CONSTRUCTION: THE NEW ERA.

### XIII. The Enlargement of Small Hospitals.

A problem which often confronts the modern architect is that of the enlargement of a cottage hospital or of a hospital of small dimensions which finds itself faced with a larger clientèle of patients than it was originally built for. It is a problem which should not be encountered with a light heart. In fact it is not too much to say that in many cases it is a problem which the promoters ought to have the courage to abandon. It is obvious that if a genuine cottage hospital has been carefully and cleverly designed to fulfil small needs on a small and compact scale its very fitness for its small intentions incapacitates it for the performing of large functions. There are, of course, cases where the mere extension of existing wards may be effected without loss or damage to the functional value of the administrative portions of the building; there may be other cases where the original designer has had his eye on the possibility of future extension, and in such rare cases all may be well. But in the vast majority of instances the plan of a small hospital will be found almost to defy extension into a useful building of larger dimensions unless, indeed, certain considerations are kept in mind.

### Readaptation *v.* Demolition.

Obviously in these days of overwhelmingly high prices it is rash to pull down any building which is reasonably sound. Therefore the architect who is called in to advise on an extension will bear in mind the grave necessity of making use in his design of every possible scrap of existing building. But, if one comes to think the matter over, it is abundantly clear that if the original small hospital was cleverly designed its very cleverness and suitability defy the idea of extension. Its compactness, its ingenious propinquity of all the parts to one another, its adequacy, so to speak, which was a neatly-balanced adequacy, all tend to render impossible the addition of greater accommodation. Even if the increased number of patients can be housed by merely lengthening the wards, the problem is only half solved by such lengthening. More patients mean more nurses and more servants —probably also a larger kitchen. It thus follows that the administrative portions of the structure must expand with the wards—and again, if, as sometimes happens, the easiest method of providing patient accommodation is to add an upper story to existing wards, the first accompanying need will be a lift, and a good-sized lift capable of conveying a recumbent case with an attendant makes sad havoc of the plan and probably displaces features which have to find a home elsewhere. It is with a view to helping those who are contemplating an enlargement of this kind that we have put together these notes based upon a varied experience in extensions of this particular kind.

First of all the promoters of such a scheme should very earnestly consider whether an enlargement is either necessary or desirable. The patients perhaps are clamouring for admission in ever increasing numbers. Still, there may be a better way out of the trouble than by additions to the existing building. Sometimes a structure which has become inadequate as a hospital proper may be made excellent as a convalescent home. In that case, and if such a convalescent home would be of use to the institution, the difficulty will be got over neatly and without waste by building a new hospital on a fresh site or on a spare portion of the existing site and adapting the existing fabric without great structural alterations to the purposes of the convalescents. Or again, it might well be that the old buildings would serve as a paying patients' block. In other cases it might become a nurses' home. In fact there are various uses connected with the institution's work to which the old building might be put almost without fundamental change.

It is therefore well at the outset to consider whether change of purpose rather than change of form will not give the most profitable life to the bricks and mortar of the old building.

### The Architect's Problem.

But in the majority of cases the promoters will very likely conclude that their needs and their funds will not permit of the abandonment of the whole of their present building to a subsidiary purpose. It is in such instances that the skill of their architectural adviser will be most severely taxed. He will, probably, if he is wise, take the plan of the existing buildings and of the available site, and consider it without any reference whatever to the existing names and allocations of the various rooms. In fact, he should make himself a tracing from which all names are omitted. He will then clear his mind as far as possible from the present associations of the bricks and mortar, and regard the whole fabric as merely so much available walling, flooring, and roofing. Thus only will he get a solution of his problem if a solution is possible. In a recent case, it was found that by a total abandonment of all the wards as wards, the problem of addition was comparatively simple. The wards, it is true, were small wards which made the problem easier and less wasteful than if they had been of normal type and size, but it so happened that the almost total reallotment of the rooms in the original structure solved the whole difficulty with neatness and without waste. Hardly a wall was destroyed, rooms which served their old purposes ill met their new duties adequately, and the only sacrifice made—the abolition of the inconvenient main staircase—was the last touch which pulled everything into shape.

This was an almost unexpected and undeserved

Previous articles appeared on Jan. 25, Feb. 8, March 1, 15, 29, April 19 May 17, June 14, July 19, Aug. 23, Sept. 20 and Oct. 25, p. 77.

**Hospital Construction : The New Era**—(*cont.*).

triumph. There are cases in which it is found virtually impossible to achieve success without some measure of distruction. The great point is to choose the sacrifice wisely.

In this connection the greatest difficulty naturally centres round the sanitary blocks and the operating-room. Unhappily,, the latter is very likely to fall a victim. It is very frequently found that some fault of lighting or of arrangement or of surroundings renders this little surgery unworthy of perpetuation. In many cases, however, it is the one feature of the hospital which is considered by its owners incontestably up to date. It is often the gift of personal generosity and is perhaps the one really new building in the whole hospital. Unfortunately, however, we constantly find it injudiciously placed. It is frequently so arranged that it is impossible to add an anæsthetising room or a sterilising room to it and constantly its lighting is insufficient or injudicious. If it has none of these faults, its position may still be a fatal bar to the transformation of functions which the architect has in view. Mercifully it is often the equipment which is the most valuable part of such an installation, and this can be moved triumphantly into new quarters.

The sanitary blocks present their own special difficulty. If a ward is being abandoned as a ward, and turned into something else it may be difficult to find a convenient function for its sanitary wing; but even this difficulty is surmountable, and as it very often happens that the sanitary group of a fairly elderly building is designed on obsolete lines the loss of this little structure as a sanitary block is often more apparent than real.

One word on drainage. If a hospital has been recently supplied with a new, sound and up-to-date drainage system it may be very unwise not to take it into account as a valuable asset, for drainage, like other builder's work, is very costly to-day. The architect must therefore be careful (in his endeavours to take a wide and unbiased view of the whole situation) to make sure whether the drainage system really is a worthy one, and, if it is, to make the best use of it that can be made without detriment to the general utility of the entire new scheme.

And again one last word. It is easy in making additions to produce a crowded building. This may lead to serious failure. Let it be always remembered that air and light, specially fresh air and sunshine, are the cheapest and sometimes the most valuable drugs in the pharmacopœia. To save them costs nothing; to lose them is to lose much.

